# Large Language Models for Diagnosis and Prognosis of Chronic Liver Diseases: A Systematic Review

**DOI:** 10.1002/hsr2.72476

**Published:** 2026-05-03

**Authors:** Basile Njei, Yazan A. Al‐Ajlouni, Abisola Ajayi, Farah Shahin, Omar Al Ta'ani, Sarpong Boateng, Gyanprakash Ketwaroo, Petr Protiva

**Affiliations:** ^1^ Engelhardt School of Global Health and Bioethics Euclid University Bangui Central African Republic; ^2^ Section of Digestive Diseases, Department of Medicine Yale University New Haven Connecticut USA; ^3^ VA Connecticut Healthcare West Haven Connecticut USA; ^4^ Ohio University Heritage College of Osteopathic Medicine Athens Ohio USA; ^5^ Yale Liver Center Yale New Haven Health New Haven Connecticut USA; ^6^ Yale International Medicine Program Yale University New Haven Connecticut USA; ^7^ Department of Rehabilitation Montefiore Medical Center New York USA; ^8^ Department of Medicine Morehouse School of Medicine Atlanta Georgia USA; ^9^ School of Medicine Al Balqa' Applied University Salt Jordan; ^10^ Department of Medicine Allegheny Health Network Pittsburgh Pennsylvania USA; ^11^ Department of Medicine Yale New Haven Health, Bridgeport Hospital Connecticut USA

**Keywords:** artificial intelligence, chronic liver disease, hepatology, large language models, systematic review

## Abstract

**Background and Aims:**

Chronic liver disease (CLD) affects more than 800 million people worldwide and remains a leading cause of morbidity and mortality. Artificial intelligence (AI), particularly machine learning, has been applied to hepatology for diagnostic and prognostic purposes. Large language models (LLMs) represent a new generation of AI with unique capabilities for processing unstructured clinical text, integrating multimodal inputs, and facilitating patient communication. Their role in CLD, however, has not been systematically reviewed.

**Methods:**

This systematic review was conducted in accordance with PRISMA guidelines and registered with PROSPERO (CRD420250650268). A literature search of five databases was performed using predefined keywords related to LLMs and CLD. Eligible studies included articles reporting diagnostic, prognostic, clinical decision support, or patient education applications of LLMs in CLD.

**Results:**

A total of 18 studies published between 2023 and 2025 met the inclusion criteria. Studies spanned multiple regions, including the USA, Europe, China, South Asia, and Australia, and employed diverse designs. Evaluated models included ChatGPT‐3.5/4, GPT‐4o, Bard, Gemini, vision‐enabled GPT, and retrieval‐augmented frameworks. Applications clustered into four thematic domains: (1) diagnostics, including HCC detection from CT/MRI, CEUS LI‐RADS classification, fibrosis staging from pathology text and histology, and MASLD identification from clinical/lab data; (2) prognosis, including cirrhosis phenotyping and fibrosis progression; (3) clinical decision support, with RAG‐based systems improving HCV guideline interpretation and agent‐based approaches generating guideline‐concordant prescriptions; and (4) patient education, where LLMs achieved 70%–90% accuracy in HBV, MASLD, cirrhosis, and AIH queries, though readability and complexity limited patient‐facing utility.

**Conclusions:**

LLMs show promising applications across the CLD spectrum, from diagnostics and prognostics to decision support and patient engagement. Current evidence is preliminary, largely retrospective, and heterogeneous. Rigorous prospective studies and careful integration strategies are required to ensure safe, effective, and equitable deployment in hepatology.

## 1 Introduction

Chronic liver disease (CLD) is a major and growing global health concern, affecting more than 800 million people and causing nearly 2 million deaths each year [1]. Its increasing prevalence is largely driven by chronic viral hepatitis (B and C), alcohol‐related liver disease, and particularly metabolic dysfunction‐associated steatotic liver disease (MASLD), which has become the leading cause of liver disease in many regions due to the worldwide obesity epidemic and related metabolic disorders [2, 3, 4]. A significant challenge in managing CLD is its often silent progression; early disease stages can be asymptomatic or present with normal liver enzyme levels, resulting in delayed diagnoses that occur only once advanced fibrosis or cirrhosis has developed [5, 6]. This late detection contributes to high morbidity, mortality, and healthcare costs, with complications such as hepatocellular carcinoma (HCC) imposing substantial clinical and economic burdens [7, 8]. Consequently, early detection and personalized management approaches—including non‐invasive fibrosis assessment tools like transient elastography and interventions targeting modifiable risk factors—are critical for improving outcomes and mitigating disease progression [9, 10, 11, 12].

In response to these clinical challenges, artificial intelligence (AI) technologies have increasingly been applied to the diagnosis and management of chronic liver diseases. Systematic reviews have demonstrated that AI, particularly through machine learning algorithms, can significantly enhance diagnostic accuracy by integrating with imaging modalities such as ultrasonography and elastography to detect liver fibrosis and MASLD more effectively [13]. Machine learning models have also leveraged genetic markers, biochemical parameters, and serum indices to predict advanced liver fibrosis and stratify patient risk, providing clinicians with more nuanced prognostic insights than traditional methods alone [14, 15, 16]. Despite these advancements, the focus has largely been on traditional AI and machine learning techniques without specific attention to large language models (LLMs), which represent a distinct and rapidly evolving branch of AI with unique capabilities particularly relevant to healthcare.

LLMs, based on transformer architectures introduced in 2017, represent a paradigm shift in natural language processing by enabling sophisticated generation and comprehension of human language [17, 18]. Unlike conventional NLP tools that rely on rule‐based or classical machine learning approaches, LLMs can process vast amounts of unstructured textual data, such as clinical notes, research literature, and patient communications, making them especially valuable for complex clinical domains. Their applications in healthcare extend beyond text analysis to include multimodal capabilities that integrate text, images, and audio, potentially enhancing diagnostic precision and patient interaction [19]. Within chronic liver disease, LLMs have shown promise in extracting relevant clinical information, supporting clinical decision‐making, and personalizing treatment recommendations by synthesizing diverse data sources [20, 21]. Despite these exciting developments, the adoption of LLMs in routine clinical practice remains in its infancy, and their distinct contributions relative to other AI technologies are yet to be systematically characterized.

The integration of LLMs into clinical workflows, particularly for chronic liver disease, faces several hurdles. Model limitations such as high error rates, potential for biased outputs, and the dynamic and heterogeneous nature of clinical data necessitate continuous refinement and rigorous validation of LLMs against real‐world patient outcomes [22, 23]. Ethical considerations related to accountability, transparency, and patient safety are paramount as these models gain traction in healthcare [24]. Given the rapid evolution of LLM technology and its unique potential in CLD management, there is a pressing need to synthesize current evidence through a focused, systematic review. Such an analysis would clarify the current state of LLM applications, identify knowledge gaps, and guide future research efforts aimed at harnessing LLMs' capabilities to improve clinical care for patients with chronic liver disease.

## 2 Methods

A systematic review was conducted in accordance with the PRISMA (Preferred Reporting Items for Systematic Reviews and Meta‐Analyses) guidelines [25]. A single literature search was performed in PubMed, Medline, Embase, CINAHL, and Scopus on July 22, 2025 using a predefined set of keywords relevant to large language models and chronic liver disease. The full search string is provided in Supporting Information S1: Table S1. The protocol related to this systematic review is registered with PROSPERO (CRD420250650268).

### 2.1 Search Strategy and Selection Criteria

Articles retrieved through the database search were imported into EndNote/Zotero reference manager software, and duplicates were removed. Titles and abstracts were independently screened by two reviewers (F.S and A.A), with full texts retrieved for all potentially eligible studies. Discrepancies were resolved by discussion with a third reviewer (Y.A.A).

Studies were eligible if they: (1) were published in the English language; (2) focused on the use of LLMs in CLD contexts, including diagnostic, prognostic, clinical decision support, or patient education applications; (3) presented primary research, pilot evaluations, or framework development; and (4) had full‐text availability. Exclusion criteria are summarized in Supporting Information S1: Table S2 and included editorials or perspectives without empirical or framework‐based evaluation, studies not involving LLMs, and non‐chronic liver disease contexts.

### 2.2 Data Extraction and Presentation

A standardized data extraction sheet (Microsoft Excel) was developed by the investigators. Data extraction was independently performed by two reviewers (F.S and A.A) and cross‐verified. Extracted variables included: study aims, design, geographic context, population/sample size (if applicable), LLM model used, clinical focus area (e.g., fibrosis, MASLD, HCC, HBV/HCV), key outcomes, and limitations. Any disagreements were resolved by discussion with a third reviewer (Y.A.A).

Extracted data are presented in tabular format and synthesized narratively in Section Results, 3. Studies were grouped by thematic domains based on their primary application of LLMs: diagnostic, prognostic, clinical decision support, and patient education. A quantitative summary of study characteristics (e.g., geographic distribution, study design, sample sizes, LLMs utilized) is also reported as part of the tabular format.

### 2.3 Quality Appraisal

Given the heterogeneity of included studies, formal quality assessment was applied selectively. Diagnostic accuracy studies were appraised with the QUADAS‐2 framework, observational studies with the CASP cohort study checklist, and conceptual or editorial pieces were narratively evaluated for methodological rigor and clarity.

### 2.4 Ethical Approval

Ethical approval was not applicable, as the review utilized data from previously published literature. All included studies reported ethics approval by their primary investigators where relevant.

### 2.5 Role of Funders

This study was not sponsored nor funded.

## 3 Results

### 3.1 Study Selection

**Figure 1:**
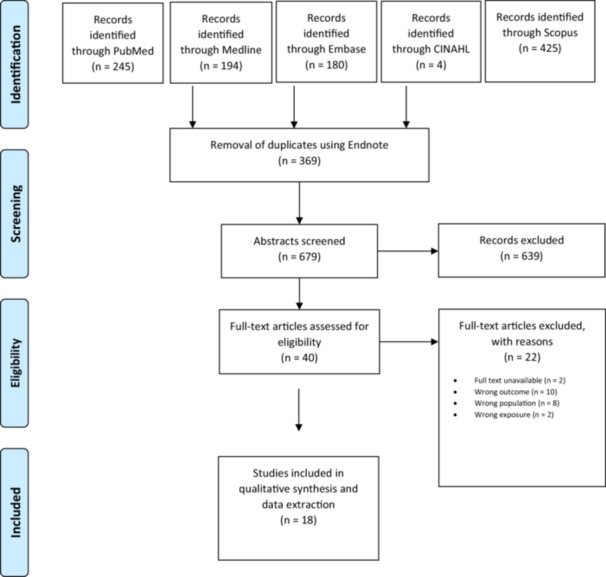
Preferred reporting items for systematic reviews and meta‐analyses (PRISMA) study selection flow diagram outlining the literature review process when searching for articles on various databases.

The initial search across five databases (PubMed, Medline, Embase, Scopus, and CINAHL) yielded a total of 1048 records (PubMed, *n *= 245; Medline, *n *= 194; Embase, *n *= 180; Scopus, *n *= 425; CINAHL, *n *= 4). After removal of 369 duplicates, 679 records remained for title and abstract screening. Of these, 639 were excluded for not meeting eligibility criteria. Forty full‐text articles were assessed, with 22 subsequently excluded (full text unavailable, *n *= 2; wrong outcome, *n *= 10; wrong population, *n *= 8; wrong exposure, *n *= 2). Ultimately, 18 studies met the inclusion criteria and were included in the qualitative synthesis and data extraction. The PRISMA flow diagram summarizing the study selection process is shown in Figure 1.

### 3.2 Study Characteristics

Table 1 demonstrates the results of our literature search and the details of extracted data for this systematic review. Across 18 records (spanning the years 2023–2025), studies were conducted in the USA [26, 27], China [28, 29, 30] (including China in multicounty cohorts [31, 32], and Europe, for example, France [31, 33], Germany [34], with a notable concentration in Italy [33, 34, 35, 36], alongside Australia [37], Sri Lanka [38], India [39], Singapore [40], and multicounty collaborations (e.g., China/France/USA [31]; Italy/Thailand/France [33]; Europe and USA [41]; Europe [42]).

**Table 1:** Applications for large language models in hepatology and hepatocellular carcinoma: Collected evidence from systematic review of literature.

First author, year	Location	Design	Sample/data source	LLM used	CLD—related focus	Main findings	Key limitations	Study Aims
Berry, 2025	USA and India	Conceptual framework	N/A	ChatGPT‐4, others	Hepatitis C as a representative of CLD.	Proposed a structured framework for integrating LLMs into gastroenterology research, highlighting potential in literature review automation, data analysis, and clinical decision support. Stressed ethical use, transparency, and interdisciplinary collaboration.	No empirical data; no HCC‐specific evaluation; framework not validated; potential bias and misinformation risks; privacy/security issues.	Present a structured framework for integrating large language models into gastroenterology, using Hepatitis C treatment as an example, with defined steps to ensure accuracy, safety, and clinical relevance while mitigating AI‐related risks. The process covers goal setting, multidisciplinary collaboration, data preparation, model development, EHR integration, real‐world validation, and continuous improvement.
Bhala, 2024	Australia and USA	Comparative pilot	4 models tested	GPT‐4o (RAG), custom GPT	ALD as a representative of CLD.	GPT‐4o achieved 14/15 correct clinical answers, close to expert (15/15) and outperforming fellow and custom GPT. Language affected performance—better in English. LLMs showed promise for staging and prognosis, but weaker in judgment‐based questions.	Small, non‐representative data sets; potential spectrum bias; limited real‐world validation; risk of amplifying training‐data biases.	Explore how LLMs can support the diagnosis, early detection, and personalized management of ALD, while emphasizing responsible integration, ethical considerations, and evidence‐based real‐world application in healthcare.
Colapietro, 2025	Italy	Cross‐sectional	4 pts, 11 raters	ChatGPT‐4	Autoimmune hepatitis as a representative of CLD.	Reliable for answering autoimmune hepatitis questions, rated well on correctness, completeness, clarity, and guideline consistency.	Very small sample; English‐only; static model; subjective scoring; no HCC or broader hepatology focus.	To assess the accuracy, completeness, comprehensiveness, and safety of ChatGPT‐4 responses to patient questions about autoimmune hepatitis.
Far, 2025	USA	Retrospective	3788 discharge summaries	GPT‐4	Assessing the accuracy of LLMs, specifically GPT‐4, in identifying cirrhosis and its complications compared to traditional code‐based methods in CLD research.	High accuracy (87.8%–98.8%) in identifying cirrhosis from text versus chart review; moderate PPVs for complications. Potential to streamline code‐based classification.	No HCC evaluation; static model; English‐only; no prospective workflow testing.	To compare the positive predictive value (PPV) of GPT‐4‐based classification with code‐based classification and manual chart review in identifying cirrhosis and its complications.
Giuffre, 2025	Europe	Agent framework	HCV patients	GPT‐4	Developing an automated treatment prescription system for patients with Chronic Hepatitis C, leveraging LLM–based agent frameworks to optimize management of CLD.	Accurate, guideline‐consistent HCV treatment recommendations; feasible for automation in hepatology.	Mostly retrospective/simulated; limited detail on complex cases; ethical and acceptance issues; no HCC assessment.	To design and evaluate an LLM agent‐based framework that can automatically generate guideline‐concordant treatment prescriptions for patients with chronic HCV infection.
Huang, 2024	China	Retrospective diagnostic	403 high‐risk pts	GPT‐4	Diagnosis of small (≤ 20 mm) HCC in high‐risk CLD patients using imaging.	High concordance with radiologists in CEUS LI‐RADS categorization for small nodules; potential for early HCC detection in surveillance.	Retrospective; depends on text report quality; challenges with atypical lesions; needs multicenter validation.	To evaluate the performance of LLMs integrated with CEUS LI‐RADS in accurately diagnosing small HCC in high‐risk patients, comparing their sensitivity and specificity to human readers.
Kresevic, 2024	Europe and USA	Development study	Guidelines data set	GPT‐4	Management of chronic HCV infection in patients with CLD.	Retrieval‐augmented generation improved accuracy and relevance of hepatology guideline interpretation versus zero‐shot prompting.	Limited to specific guidelines; no patient data; generalizability depends on database quality; no real‐world testing.	To evaluate how integrating LLMs with structured medical guidelines can improve clinical decision support systems (CDSSs) for chronic HCV management, focusing on accuracy and reliability of guideline interpretation.
Laohawetwanit, 2025	Italy	Diagnostic accuracy	150 MASLD biopsies	ChatGPT‐4	Assessment of liver fibrosis staging in metabolic dysfunction‐associated steatohepatitis.	Strong agreement with pathologists in fibrosis staging; particularly accurate in identifying advanced fibrosis.	Text‐based pathology interpretation only; retrospective; variability in report style may affect performance.	To evaluate the diagnostic accuracy of ChatGPT‐4‐vision in staging liver fibrosis from histopathological images and compare its performance to expert liver pathologists, including the impact of in‐context learning on model accuracy.
Li, 2025	China	Cross‐sectional	HBV Q&A	ChatGPT‐3.5, 4.0, Gemini	Management and patient education of chronic HBV infection.	GPT‐4.0 achieved highest objective (80.8%) and subjective scores; excelled in diagnosis questions.	No real patient interaction; evaluator bias possible; readability > 8th grade; no HCC focus.	To evaluate and compare the accuracy and readability of responses provided by ChatGPT‐3.5, ChatGPT‐4.0, and Google Gemini when answering HBV‐related questions, assessing their potential as adjunct informational tools for patients and physicians.
Niriella, 2025	Sri Lanka	Cross‐sectional	150 FAQs	Early ChatGPT, Bard	Evaluation of freely accessible, general‐purpose LLMs in providing patient information on liver disease.	70%–85% correct responses; accuracy dropped with more technical Qs; readability often too high for patients.	No patient testing; baseline models; subjective scoring; limited to written FAQs.	To assess the accuracy, completeness, and quality of patient information generated by general‐purpose LLMs for frequently asked questions on liver disease, determining their reliability as a source of information for patients.
Panzeri, 2025	Italy, Thailand, France	Retrospective	59 biopsies	ChatGPT‐4 vision	Staging of liver fibrosis in metabolic dysfunction‐associated steatohepatitis.	Comparable accuracy to expert pathologists with selected images (81%); improved to 88% with in‐context learning; lower accuracy with random fields (54%).	Small sample; performance image‐selection dependent; weaker at F4 staging without context.	To evaluate the diagnostic accuracy of ChatGPT‐4‐vision in assessing liver fibrosis from histopathological images and compare its performance with expert liver pathologists, including the effect of in‐context learning on accuracy.
Sheng, 2025	China, France, USA	Retrospective	1200 liver lesion pts	GPT‐4	Diagnosis of FLLs in patients with CLD using imaging.	Diagnostic accuracy (87%) close to radiologists (92%); HCC sensitivity 85%, specificity 88%; substantial inter‐rater agreement.	Retrospective; text‐based report analysis only; tertiary‐center bias; needs prospective testing.	To evaluate the diagnostic accuracy of ChatGPT‐4o and Gemini in interpreting CT/MRI reports for FLLs, comparing their performance with radiologists of different experience levels and assessing any incremental value when combined with human interpretation.
Wu, 2025	China	Cross‐sectional	450 MASLD pts	GPT‐4	Diagnosis of MASLD in CLD patients.	MASLD diagnosis accuracy 82%, comparable to FibroScan; integrated clinical and lab data for staging.	Retrospective; input‐dependent; moderate sample; no longitudinal outcomes.	To evaluate the diagnostic performance of GPT‐3.5, GPT‐4, and GPT‐4V for MASLD using textual data and ultrasound images, comparing their accuracy to traditional risk scores like FLI and USFLI.
Yeo, 2023	USA	Cross‐sectional	164 Qs on cirrhosis/HCC	ChatGPT‐3.5	Management and patient education for cirrhosis and HCC in CLD patients.	~74% correct HCC answers; better for general than complex clinical Qs.	Outdated info risk; no real‐time clinical testing; preset Qs only.	To evaluate the accuracy, comprehensiveness, and reproducibility of ChatGPT in answering questions on cirrhosis and HCC, including its capacity to provide emotional support to patients and caregivers.
Zhang, 2025	China, Singapore, USA	Cross‐sectional	Fatty liver vignettes	ChatGPT‐4, Bard	Grading of fatty liver disease severity using general‐purpose LLMs.	ChatGPT‐4 slightly better at nuanced reasoning than Bard.	No metrics; small vignette‐based study; general‐purpose models.	To compare the diagnostic accuracy of ChatGPT‐4 and Google Bard in grading non‐alcoholic fatty liver disease (NAFLD) based on histological images.
Pugliese, 2024 (study)	Italy	Cross‐sectional	120 MASLD Qs (Italian)	GPT‐4	Patient counseling and education for MASLD.	High accuracy (~88%) and clarity in patient counseling content; useful for education.	Limited to Italian; MASLD‐only focus; written responses only.	To evaluate the accuracy, completeness, and comprehensibility of ChatGPT‐3.5 in providing MASLD‐related counseling to Italian‐speaking patients and to assess whether language affects its performance.
Pugliese, 2025 (editorial)	Italy, Germany, USA	Editorial	N/A	GPT‐3.5, 4	The application of LLMs in the context of MASLD, exploring their potential role in enhancing clinical practice through generative artificial intelligence.	Summarized MASLD‐related LLM opportunities and challenges.	No original research data.	To evaluate the integration of LLMs into clinical practice for MASLD, examining their impact on diagnosis, patient counseling, and overall management.
Giuffrè, 2025 (editorial)	Italy, USA	Editorial	N/A	ChatGPT	Use of ChatGPT and large language models in gastroenterology, including management and patient education related to CLD.	Discussed need for standardized AI evaluation in gastroenterology.	No empirical validation; general commentary.	To critically evaluate the accuracy, reliability, and privacy considerations of ChatGPT applications in gastroenterology, advocating for methodological rigor to ensure safe and effective integration into clinical practice.

Abbreviations: AI, artificial intelligence; ALD, alcohol‐associated liver disease; AUC, area under the receiver operating characteristic curve; CEUS, contrast‐enhanced ultrasound; CPA, collagen proportionate area; CT, computed tomography; FLL, focal liver lesion; FAQ, frequently asked question; FOV, field of view; GPT, generative pre‐trained transformer; HBV, hepatitis B virus; HCC, hepatocellular carcinoma; HCV, hepatitis C virus; LI‐RADS, liver imaging reporting and data system; LLM, large language model; MASLD, metabolic dysfunction‐associated steatotic liver disease; MASH, metabolic dysfunction‐associated steatohepatitis; MRI, magnetic resonance imaging; NIT, non‐invasive test; PPV, positive predictive value; RAG, retrieval‐augmented generation.

Designs spanned cross‐sectional [27, 29, 30, 34, 35, 38, 40] (*n* = 7), retrospective [26, 28, 31, 33] (*n* = 4), diagnostic accuracy [36] (*n* = 1), comparative pilot [37] (*n* = 1), development/framework [41, 42] (*n* = 2), conceptual framework [39] (*n* = 1), and editorial/commentary [34, 43] (*n* = 2). Sample sizes and data sources ranged from very small patient cohorts (e.g., 4 patients with 11 expert raters [35]) to large text corpora (3788 discharge summaries [26]) and multicenter imaging cohorts (1200 patients [31]; 403 high‐risk patients [28]). Additional data sets included 59 digitized biopsy slides [33], ~150 MASLD biopsies [36], 450 MASLD patients [30], and question‐based corpora (e.g., 164 cirrhosis/HCC questions [27]; 150 FAQs [38]; 120 MASLD questions in Italian [34]).

LLMs included ChatGPT‐3.5 [27, 29, 34], ChatGPT‐4/GPT‐4 [26, 28, 30, 31, 35, 36], GPT‐4o and GPT‐4o with RAG [31, 37], customized GPT [37], ChatGPT‐4 vision/GPT‐4V [30, 33], Google Bard [38, 40], Google Gemini [29, 31], and RAG‐based frameworks for guideline interpretation [41] and agent‐based prescription systems [42].

In terms of health outcomes studies, and across included studies, MASLD was the most frequently studied condition (*n* = 5 [30, 33, 34, 36, 40]), followed by HCC and focal liver lesions (*n* = 2 [28, 31]), cirrhosis and its complications (*n* = 2 [26, 27]), HBV (*n* = 1 [29]), HCV (*n* = 2 [41, 42]), AIH (*n* = 1 [35]), and ALD (*n* = 1 [37]). Several conceptual or editorial pieces addressed broader gastroenterology or hepatology contexts without a single disease focus [34, 39, 42]. Furthermore, focus areas encompassed both direct HCC tasks (small HCC diagnosis with CEUS LI‐RADS [28]; HCC detection within focal liver lesion classification from CT/MRI reports [31]) and indirect CLD endpoints, including fibrosis staging from pathology text or histology [33, 36], MASLD identification and staging [30, 34, 40], cirrhosis identification and complications from discharge summaries [26], HBV knowledge and counseling [29], HCV management and guideline‐concordant treatment [41, 42], and AIH question‐answering [35]. Conceptual/editorial pieces addressed frameworks for LLM integration in gastroenterology and MASLD [34, 39, 42].

### 3.3 Quality Assessment

**Figure 2:**
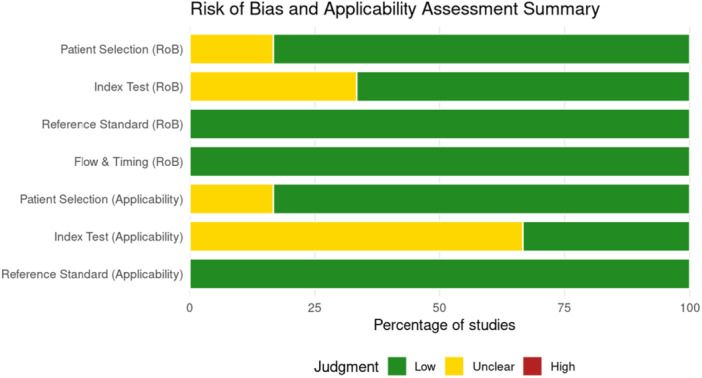
Summary of QUADAS‐2 risk of bias and applicability assessments across diagnostic accuracy studies.

**Figure 3:**
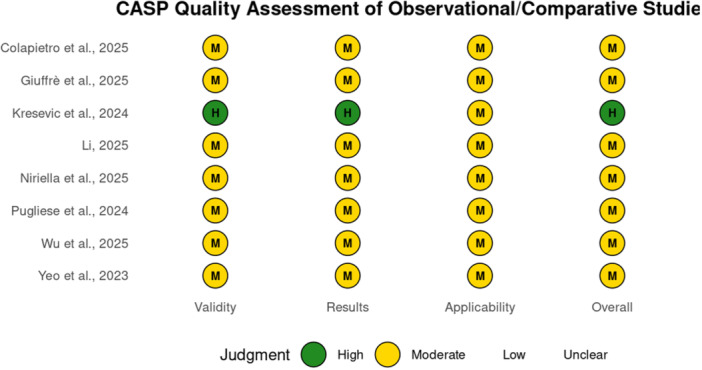
CASP quality assessment of included observational/comparative studies.

Eighteen studies were appraised using frameworks appropriate to their design. Diagnostic accuracy studies (Supporting Information S1: Table S3) demonstrated overall low risk of bias, with occasional concerns in index test and applicability domains, particularly when image selection was not standardized, or reporting was incomplete, as shown in Figure 2. Observational and comparative evaluations (Supporting Information S1: Table S4) were generally of moderate quality, with strengths including blinded expert raters and structured case designs, but common limitations such as small sample sizes, reliance on simulated or curated data, and limited generalizability beyond single countries or disease areas (Figure 3). Conceptual and editorial contributions (Supporting Information S1: Table S5) were narratively appraised and found to be methodologically transparent and highly relevant for framing future research, though lacking empirical validation.

## 4 LLM Applications in CLD: Thematic Analysis

### 4.1 Diagnostic Applications

#### 4.1.1 Liver Lesion Detection and Characterization

Several studies evaluated LLMs for diagnostic purposes across chronic liver diseases, including HCC, cirrhosis, MASLD, autoimmune hepatitis (AIH), and chronic viral hepatitis. In a multicenter retrospective study involving 1200 patients with focal liver lesions who underwent CT or MRI, GPT‐4 achieved a diagnostic accuracy of 87% compared with 92% for radiologists, with 85% sensitivity and 88% specificity for HCC detection, an F1‐score of 0.83, an AUC of 0.89, and a Cohen's *κ* of 0.75 when compared with radiologist interpretation [31]. Similarly, in a study of 403 high‐risk patients with untreated small focal liver lesions (≤ 20 mm) assessed by contrast‐enhanced ultrasound (CEUS), GPT‐4 categorized lesions according to the CEUS Liver Imaging Reporting and Data System (LI‐RADS) with accuracy, sensitivity, specificity, and inter‐rater agreement metrics that showed high concordance with expert radiologists [28].

### 4.2 Fibrosis Staging

Applications in fibrosis staging were evaluated in both text‐ and image‐based contexts. An Italian multicenter diagnostic study of approximately 150 MASLD biopsy specimens found that ChatGPT‐4 classified fibrosis stages (F0–F4) from histopathology reports with strong agreement compared to expert pathologists, particularly for advanced fibrosis (≥ F3) [36]. In another study of 59 digitized biopsy slides from patients with metabolic dysfunction–associated steatohepatitis (MASH), ChatGPT‐4 vision achieved 81% accuracy for fibrosis staging when expert‐selected fields of view (FOVs) were provided and 54% accuracy with randomly cropped FOVs. In‐context learning improved performance to 88% and 77%, respectively, with stage‐specific recall of 43% for F1, 79% for F2, 100% for F3, and 40% for F4, alongside a correlation with collagen proportionate area (*ρ* = 0.69, *p* < 0.01) [33].

### 4.3 Non‐Invasive Identification of Liver Disease and Cirrhosis

LLMs have also been applied to non‐invasive identification of liver disease from clinical and laboratory data. In a cross‐sectional study of 450 patients at risk for or suspected of MASLD, GPT‐4 achieved a diagnostic accuracy of 82%, with a sensitivity of 79%, specificity of 85%, and an F1‐score of 0.81, demonstrating performance comparable to FibroScan and traditional risk scores [30]. Another study of 3788 discharge summaries assessed GPT‐4 for cirrhosis and its complications, reporting accuracy of 87.8%–98.8% for cirrhosis detection and positive predictive values ranging from 41.7% to 72.8% for complications such as hepatic encephalopathy, ascites, and gastrointestinal bleeding [26].

### 4.4 Knowledge‐Based Diagnostic Assessment and Disease Grading

In addition to disease staging and detection, LLMs have been used to evaluate knowledge‐based diagnostic accuracy. In Italy, ChatGPT‐4 responses to 50 questions on AIH were rated highly by hepatology experts for correctness, completeness, and clarity [35]. Similarly, ChatGPT‐3.5 achieved approximately 74% accuracy in answering HCC‐ and cirrhosis‐related questions [27], while ChatGPT‐4 outperformed other models when tested on hepatitis B virus (HBV)‐related questions, with objective accuracy of 80.8% and superior subjective scores [29]. A cross‐sectional evaluation of 150 frequently asked patient questions covering liver diseases, including HCC risk, found that freely available general‐purpose LLMs such as ChatGPT and Bard produced 70%–85% correct responses, although accuracy declined with technical complexity, and readability often exceeded recommended levels for patients [38]. Finally, in a multicounty vignette‐based study, ChatGPT‐4 demonstrated more consistent grading of fatty liver disease severity compared to Bard, although neither model was specifically optimized for hepatology [32].

### 4.5 Prognostic and Risk Prediction Applications

Several studies evaluated LLMs for prognostic modeling and risk prediction in chronic liver disease, including applications in alcohol‐associated liver disease, cirrhosis and its complications, and fibrosis staging in MASLD and MASH [26, 33, 36, 37].

In a comparative pilot study of four models addressing alcohol‐associated liver disease, GPT‐4o with retrieval‐augmented generation achieved 14 of 15 correct responses, closely matching expert gastroenterologists (15/15), and outperforming a gastroenterology fellow (10/15) and a customized GPT model (8/15, improving to 10/15 when tested with English‐language input). The customized model showed reduced accuracy with non‐English inputs, and both models demonstrated lower performance on judgment‐based questions [37].

A retrospective study analyzing 3788 discharge summaries reported that GPT‐4 achieved 87.8%–98.8% accuracy in identifying cirrhosis when compared with manual chart review. Positive predictive values for complications varied, including 41.7% for hepatic encephalopathy, 72.8% for ascites, and 59.8% for gastrointestinal bleeding [26].

Fibrosis staging outcomes relevant to disease progression were also reported. In an Italian multicenter study of approximately 150 MASLD biopsy specimens, ChatGPT‐4 showed strong agreement with expert pathologists, with particularly high accuracy in detecting advanced fibrosis (≥ F3) [36]. In another study of 59 digitized biopsy slides from patients with metabolic dysfunction–associated steatohepatitis, ChatGPT‐4 vision achieved 81% accuracy in fibrosis staging with expert‐selected fields of view and 54% accuracy with randomly cropped fields. With in‐context learning, accuracy improved to 88% and 77%, respectively. Stage‐specific recall for expert‐selected fields was 43% for F1, 79% for F2, 100% for F3, and 40% for F4, with correlation to collagen proportionate area of *ρ* = 0.69 (*p* < 0.01) [33].

### 4.6 Clinical Decision Support and Guideline Interpretation

Several studies explored the use of LLMs for clinical decision support and guideline‐based applications in chronic liver disease, including treatment prescription frameworks, automated guideline interpretation, and management support [39, 41, 42].

In Europe, an agent‐based framework using GPT‐4 was developed to generate guideline‐concordant treatment prescriptions for patients with chronic hepatitis C. The system produced accurate and reliable recommendations that were aligned with established clinical guidelines, demonstrating feasibility for automation in hepatology [42]. Another study focusing on the integration of LLMs with structured clinical guidelines evaluated a retrieval‐augmented generation framework for chronic HCV management. This approach significantly improved the accuracy, relevance, and reliability of guideline interpretation when compared with zero‐shot prompting [41].

A conceptual framework was also proposed outlining how LLMs could be integrated into gastroenterology research and practice, using hepatitis C treatment as an example of chronic liver disease management. The framework detailed structured steps, including goal setting, multidisciplinary collaboration, data preparation, model development, EHR integration, real‐world validation, and continuous improvement [39].

### 4.7 Patient Education and Communication

Several studies evaluated LLMs for their ability to provide patient‐facing information and counseling across chronic liver diseases, including MASLD, HBV, cirrhosis, and HCC [27, 29, 34, 35, 38, 40].

In China, ChatGPT‐4.0 achieved the highest overall performance among three LLMs tested on HBV‐related patient questions, with objective accuracy of 80.8% and the highest subjective ratings, particularly excelling in the diagnosis domain [29]. In Sri Lanka, an evaluation of 150 frequently asked patient questions on liver disease showed that general‐purpose LLMs such as early ChatGPT and Bard produced 70%–85% correct responses, though accuracy was lower for technical queries and readability often exceeded patient‐friendly levels [38].

A US study assessing ChatGPT‐3.5 on 164 cirrhosis‐ and HCC‐related questions reported approximately 74% correct responses, with stronger performance on general questions compared with complex clinical ones [27]. In Italy, ChatGPT‐4 was evaluated on 120 MASLD‐related patient questions in Italian, achieving ~88% accuracy, 82% completeness, and 90% comprehensibility, supporting its utility for counseling in that setting [34].

Applications were also tested in autoimmune hepatitis, where ChatGPT‐4 responses to 50 clinical questions were assessed by hepatology experts and rated highly for correctness, completeness, and clarity [35]. Additionally, a multicountry vignette‐based study compared ChatGPT‐4 with Bard in grading fatty liver disease, finding that ChatGPT‐4 demonstrated greater consistency and clinical reasoning [40].

## 5 Discussion

This systematic review set out to examine the emerging role of large language models in chronic liver disease across diagnostic, prognostic, decision support, and patient‐facing domains. To date, no prior review has synthesized this literature, despite its rapid expansion in recent years. The majority of included studies were published between 2023 and 2025, reflecting the fast pace at which applications are being developed and tested. Collectively, the evidence shows that LLMs have been deployed across a diverse range of CLD contexts. Diagnostic studies featured prominently, with models such as GPT‐4 approaching radiologist‐level performance in the detection of focal liver lesions and hepatocellular carcinoma, and demonstrating high concordance with expert pathologists in fibrosis staging. Work on metabolic dysfunction–associated steatotic liver disease frequently reported accuracies in the 80%–90% range for staging and risk prediction. Prognostic applications highlighted the potential of these tools to process large clinical data sets for identifying risk factors and predicting cirrhosis‐related outcomes. In addition, several studies evaluated their use in decision support, including retrieval‐augmented frameworks to improve guideline interpretation in hepatitis C, while others explored conversational applications for patient counseling and education. Together, these findings underline both the breadth of clinical tasks addressed and the early promise of LLMs across the spectrum of chronic liver disease.

When considered by model type, several patterns emerged across the empirical studies. Across the empirical studies included in this review, GPT‐4–based models were the most frequently evaluated and generally demonstrated strong performance across a range of hepatology tasks, including question answering, text‐based diagnostic classification, imaging‐report interpretation, and fibrosis assessment. Retrieval‐augmented approaches appeared particularly helpful for knowledge‐grounded tasks such as hepatitis C guideline interpretation and alcohol‐associated liver disease clinical reasoning, while vision‐enabled GPT models showed promise in pathology‐ and imaging‐related applications. Other models, including Google Bard and Google Gemini, also demonstrated utility in selected studies, particularly for patient‐facing question answering and comparative diagnostic tasks. However, these findings should be interpreted cautiously, as comparisons were made across heterogeneous study designs, data sets, prompts, and outcome measures rather than through standardized head‐to‐head benchmarking. A summary of the LLMs evaluated in empirical studies, along with their reported task‐specific advantages and key limitations, is provided in Supporting Information S1: Table S6.

Prior work in hepatology has primarily examined artificial intelligence and machine learning approaches, with extensive research into predictive models, radiomics, and non‐invasive diagnostic tools such as FibroScan and elastography [37, 44, 45, 46, 47, 48]. While these methods have demonstrated clinical value, they were largely limited to structured data inputs or imaging features, and their outputs were constrained to narrow diagnostic or prognostic tasks. To the best of our knowledge, this review is the first to focus specifically on large language models in chronic liver disease. Earlier reviews considered LLMs as part of the broader field of natural language processing in hepatology. For example, Ubeda et al. summarized applications of NLP and LLMs for extracting and classifying features from unstructured radiology reports [45], while Omar et al. reviewed NLP and LLM approaches across gastroenterology and hepatology more broadly [44]. In both cases, LLMs were discussed within the wider trajectory of NLP development, rather than being the central focus.

The novelty of our review lies in isolating LLMs as a distinct technological advance and synthesizing their applications across hepatology. Unlike traditional ML pipelines or earlier NLP systems, LLMs extend beyond single‐task diagnostics: they have been applied to the interpretation of complex clinical text, clinical decision support through retrieval‐augmented guideline frameworks, and direct patient‐facing communication and counseling. Importantly, LLMs offer practical advantages over conventional AI approaches, including the ability to handle unstructured clinical data and perform multiple tasks without task‐specific model development, enhancing real‐world applicability. Their deployment through user‐friendly interfaces or APIs improves accessibility across diverse settings, while requiring less extensive data labeling and development effort, suggesting potential cost‐effectiveness and scalability. Whereas prior AI work in hepatology has largely centered on imaging or biopsy‐based tasks, LLMs can operate across multiple modalities, including unstructured electronic health records, radiology and pathology reports, and conversational interactions. This ability to bridge structured and unstructured data highlights a broader scope of application than that achieved by earlier diagnostic or prognostic tools.

### 5.1 Strengths and Limitations of the Evidence Base

The evidence synthesized in this review has several strengths. Studies originated from diverse geographic regions, with representation across North America, Europe, Asia, and South Asia, reflecting global early adoption of LLM applications in hepatology. A variety of LLM architectures were evaluated, including general‐purpose models (ChatGPT‐3.5, ChatGPT‐4, GPT‐4o, Bard, Gemini), vision‐enabled frameworks, and retrieval‐augmented designs. The breadth of clinical contexts covered multiple subtypes of chronic liver disease, including MASLD, cirrhosis, HCC, HBV, HCV, AIH, and ALD, emphasizing the potential versatility of these tools.

Nonetheless, the evidence base remains limited in important ways. Most studies were retrospective or cross‐sectional, with small sample sizes and few real‐world or prospective validations. Many relied on English‐language input, with performance often lower in non‐English contexts, raising concerns about generalizability. Evaluation metrics varied considerably across studies, limiting comparability and synthesis. In several instances, expert ratings of model outputs were used as the gold standard rather than patient‐level or clinical outcomes. Furthermore, external validation was sparse, and reproducibility is challenged by the pace of model development, where version updates can rapidly alter performance characteristics.

### 5.2 Clinical and Research Implications

LLMs hold promise for augmenting existing diagnostic workflows in hepatology, particularly in radiology and pathology. Models have achieved accuracy approaching that of radiologists in detecting focal liver lesions and HCC from CT/MRI reports, and high concordance in LI‐RADS classification for CEUS, suggesting opportunities for deployment as secondary readers, tools for standardizing report language, or as triage aids in high‐volume settings [28, 31]. In pathology, both text‐based and vision‐enabled LLMs demonstrated strong agreement with expert pathologists in fibrosis staging, with performance influenced by field‐of‐view selection and enhanced by in‐context learning. These applications could reduce inter‐observer variability, support quality assurance, and expedite turnaround times in histopathology workflows [33, 36].

Beyond diagnostics, LLMs demonstrated utility in large‐scale chart review and registry development. In a study of nearly 4000 discharge summaries, GPT‐4 classified cirrhosis with high accuracy and identified complications such as hepatic encephalopathy and ascites with moderate predictive values [26]. Such applications highlight the potential for automated phenotyping, enabling the construction of disease registries and surveillance cohorts at scale. Similarly, models trained on routine clinical and laboratory data achieved accuracies comparable to FibroScan in identifying MASLD, demonstrating feasibility for risk stratification and population health management [30]. Prognostic use cases were also illustrated in ALD, where comparative evaluations highlighted task‐ and language‐dependent variability, exemplifying the importance of tailoring inputs to maximize predictive performance [37].

For clinical decision support, retrieval‐augmented frameworks demonstrated improved accuracy and reliability in guideline interpretation for chronic HCV, while agent‐based architectures successfully generated treatment recommendations consistent with published guidelines. These approaches point toward near‐term opportunities for embedding auditable, version‐controlled decision support tools into electronic health records, where outputs can be linked directly to clinical workflows [41, 42].

Patient‐facing applications were tested across multiple liver diseases, including HBV, MASLD, cirrhosis, AIH, and HCC. Studies consistently reported moderate to high accuracy in answering patient questions, with performance ranging from ~70% to 90% depending on topic and complexity [27, 29, 34, 35]. While these findings support supervised use of LLMs as adjuncts to counseling and education, concerns remain regarding readability, the handling of technical or complex queries, and risks of misinformation if models are deployed without safeguards.

Equity considerations are critical. Several studies highlighted performance degradation in non‐English contexts or when models were queried in languages other than English, raising concerns that early deployments may reinforce disparities in care access and quality [29, 37, 38]. Ensuring inclusivity will require systematic evaluation across languages and health literacy levels, as well as deliberate adaptation of outputs for diverse patient populations.

For research, these early applications highlight the need for prospective, multicenter studies that link LLM outputs to clinical outcomes such as diagnostic accuracy, treatment initiation, and patient safety. Standardized evaluation metrics should be adopted to allow comparability across studies, while reporting of prompt design, model version, and dataset characteristics is needed to enhance reproducibility. In multimodal applications, protocols should address image selection strategies and contextual inputs given demonstrated variability in performance. As models continue to evolve rapidly, mechanisms for ongoing audit, version control, and external validation will be essential to ensure safety, transparency, and clinical trustworthiness.

### 5.3 Future Directions

Future work should move beyond retrospective and vignette‐based studies toward prospective, multicenter validation of LLMs across diverse clinical settings. These studies should evaluate not only agreement with expert interpretation but also downstream clinical outcomes such as diagnostic yield, treatment initiation, patient safety, and workflow efficiency. Multimodal applications that integrate imaging, histopathology, laboratory data, and clinical text remain largely untested in hepatology but represent a critical next step to mirror real‐world practice. Equally important is the development of standardized evaluation frameworks and reporting guidelines to enable comparability across studies and ensure reproducibility as models evolve.

Beyond research, future directions should focus on thoughtful clinical integration. Embedding LLMs into radiology, pathology, and EHR systems requires attention to governance, transparency, and auditability, with mechanisms for version control and continuous performance monitoring. Efforts must also address equity, including language accessibility and plain‐language adaptation for patient‐facing tools, to avoid reinforcing disparities in care. Collaboration between clinicians, data scientists, ethicists, and regulatory bodies will be essential to ensure that LLMs advance from proof‐of‐concept toward safe, effective, and equitable tools in hepatology.

### 5.4 Limitations of This Review

This review also has methodological limitations that should be acknowledged. Only a single database was searched, which may have led to the omission of relevant studies indexed elsewhere. Restriction to English‐language publications introduces the possibility of language bias, particularly important given that LLM performance varies by language. Heterogeneity across study designs and reported outcomes precluded formal meta‐analysis, meaning that quantitative synthesis was not feasible. The possibility of publication bias should also be considered, as studies demonstrating positive or novel applications of LLMs are more likely to be published than negative or null findings. Finally, given the rapid evolution of LLM technologies, with frequent model updates and new architectures emerging, the findings of this review represent a snapshot in time and may become outdated quickly.

## 6 Conclusion(s)

LLMs demonstrate promising applications across the spectrum of CLDs, with studies highlighting their potential in diagnosis, prognosis, clinical decision support, and patient engagement. The current evidence base, however, is preliminary, largely retrospective, and heterogeneous, with limited external validation. As these tools continue to evolve rapidly, rigorous prospective evaluations and careful strategies for integration into clinical practice are required to ensure their safe, effective, and equitable use in hepatology.

## Author Contributions

**Basile Njei:** conceptualization, methodology, supervision, project administration, writing – review and editing. **Yazan A. Al‐Ajlouni:** conceptualization, methodology, writing – original draft, writing – review and editing, project administration, visualization. **Abisola Ajayi:** methodology, writing – review and editing. **Farah Shahin:** writing – original draft, writing – review and editing. **Omar Al Ta'ani:** conceptualization, methodology, writing – review and editing, writing – original draft. **Sarpong Boateng:** methodology, writing – review and editing. **Gyanprakash Ketwaroo:** conceptualization, writing – original draft. **Petr Protiva:** writing – review and editing, methodology.

## Funding

The authors have nothing to report.

## Conflicts of Interest

The authors declare no conflicts of interest.

## Transparency Statement

The lead author, Basile Njei, affirms that this manuscript is an honest, accurate, and transparent account of the study being reported; that no important aspects of the study have been omitted; and that any discrepancies from the study as planned (and, if relevant, registered) have been explained.

## Supporting information

Supporting File

## Data Availability

The data that support the findings of this study are available on request from the corresponding author. The data are not publicly available due to privacy or ethical restrictions.
