# Supplementary material for: Large Language Models for Diagnosis and Prognosis of Chronic Liver Diseases: A Systematic Review
Source: Health Sci Rep. 2026 May 3;9(5):e72476. doi: 10.1002/hsr2.72476 (PMC13136603; doi:10.1002/hsr2.72476)
Supplement: Supplementary file 1 — Supporting File [file HSR2-9-e72476-s001.docx]

**Table S1.** Search Concepts and Corresponding Keywords for the Systematic Review

| Concept | Keywords |
| --- | --- |
| \| LLMs \| \| --- \|  \|  \| \| --- \| | "large language model*" OR "LLM*" OR "transformer model*" OR "GPT*" OR "BERT" OR "BioBERT" OR "ClinicalBERT" OR "Med-PaLM" OR "LLaMA" OR "PaLM" OR "foundation model*" OR "generative AI" |
| \| Diagnosis & Prognosis \| \| --- \|  \|  \| \| --- \| | "diagnos*" OR "prognos*" OR "risk prediction" OR "clinical prediction model*" OR "risk stratification" OR "outcome prediction" OR "mortality prediction" OR "disease progression" OR "clinical decision support" OR "CDSS" |
| \| CLD \| \| --- \|  \|  \| \| --- \| | "chronic liver disease*" OR "CLD" OR "liver fibrosis" OR "liver cirrhosis" OR "cirrhosis" OR "hepatic fibrosis" OR "NAFLD" OR "MASLD" OR "MASH" OR "alcoholic liver disease" OR "ALD" OR "viral hepatitis" OR "hepatitis B" OR "hepatitis C" OR "autoimmune hepatitis" OR "primary biliary cholangitis" OR "PBC" OR "primary sclerosing cholangitis" OR "PSC" OR "Wilson's disease" OR "hemochromatosis" |

**Table S2.** Inclusion and Exclusion Criteria for Study Selection

| Category | Inclusion Criteria | Exclusion Criteria |
| --- | --- | --- |
| Population | Studies involving patients with chronic liver diseases, including MASLD, MASH, ALD, viral hepatitis, autoimmune liver diseases, and genetic/metabolic liver disorders. | Studies focusing exclusively on acute liver conditions or non-liver diseases. |
| Intervention | Studies assessing large language models for diagnosis, prognosis, risk stratification, or clinical decision support in chronic liver diseases. | Studies that do not involve large language models or focus only on general AI/ML models. |
| Comparator | No restrictions | |
| Outcomes | Studies reporting performance metrics, clinical integration, or comparative effectiveness of large language models against traditional methods. | Studies that do not assess model performance in a quantitative or qualitative manner. |
| Study Design | - Observational studies (retrospective/prospective cohort studies, cross-sectional studies evaluating model performance in chronic liver diseases).  - Interventional studies (RCTs and non-randomized trials assessing model integration into clinical practice).  - Conference abstracts and preprints with sufficient methodological detail and relevant data. | - Case reports and case series without systematic evaluation of model performance.  - Editorials, opinion pieces, and non-peer-reviewed sources.  - Studies focused solely on AI ethics, theoretical frameworks, or policy discussions without empirical evaluation.  - Animal studies and preclinical research without human data.  - Systematic reviews and meta-analyses summarizing applications in hepatology. |
| Language | Studies published in English. | Studies published in languages other than English. |
| Publication Date | No restrictions | |

**Abbreviations:** AI – Artificial Intelligence; ALD – Alcoholic Liver Disease; APRI – Aspartate Aminotransferase to Platelet Ratio Index; CDSS – Clinical Decision Support System; CLD – Chronic Liver Disease; FIB-4 – Fibrosis-4 Index; LLM – Large Language Model; MASH – Metabolic Dysfunction-Associated Steatohepatitis; MASLD – Metabolic Dysfunction-Associated Steatotic Liver Disease; MELD – Model for End-Stage Liver Disease; ML – Machine Learning; NLP – Natural Language Processing; RCT – Randomized Controlled Trial.

**Table S3.** QUADAS-2 Risk of Bias and Applicability Assessment for Diagnostic Accuracy Studies

| Study | Patient Selection (RoB) | Index Test (RoB) | Reference Standard (RoB) | Flow & Timing (RoB) | Patient Selection (Applicability) | Index Test (Applicability) | Reference Standard (Applicability) |
| --- | --- | --- | --- | --- | --- | --- | --- |
| Far, 2025 | Low | Low | Low | Low | Low | Low | Low |
| Huang, 2024 | Low | Low | Low | Low | Low | Unclear | Low |
| Laohawetwanit, 2025 | Low | Unclear | Low | Low | Low | Unclear | Low |
| Panzeri, 2025 | Low | Unclear | Low | Low | Low | Unclear | Low |
| Sheng, 2025 | Low | Low | Low | Low | Low | Low | Low |
| Zhang, 2025 | Unclear | Low | Low | Low | Unclear | Unclear | Low |

**Table S4.** CASP Quality Assessment of Prognostic/Observational Studies

| Study | Validity (Study Design & Confounders) | Results (Clarity & Precision) | Applicability (Generalizability & Relevance) | Overall Quality Judgment |
| --- | --- | --- | --- | --- |
| Colapietro et al., 2025 | Evaluation of 12 selected patient questions across three domains (diagnosis, quality of life, treatment); 11 key opinion leaders graded responses. Strength: multiple expert raters; Limitation: very small sample of questions, possible selection bias, and subjective evaluation. | Reported clear median Likert scores: accuracy 5/6, completeness 2/2, comprehensiveness 3/3, safety 5/5. Agreement among raters was very poor (Fleiss Kappa ≈ 0), reducing reliability. | Relevant to patient education and counseling in autoimmune hepatitis, but generalizability limited by single disease, small set of questions, and single chatbot version. | Moderate |
| Giuffrè et al., 2025 | Simulation with 50 synthetic HCV clinical cases (genotype, fibrosis, meds, prior therapy). Compared single vs multi-agent GPT-3.5 and GPT-4 configurations. Strengths: structured case design, systematic comparison of architectures. Limitations: synthetic rather than real patient data, no clinician adjudication of final prescriptions. | Results clearly reported with accuracy percentages across configurations (baseline, single agent, multi-agent, specialized multi-agent). Statistical testing applied (p values), showing significant improvements. No CIs reported, small case sample. | Relevant to future decision-support in HCV treatment. Limited generalizability due to artificial case design, absence of external validation, and reliance on guideline fine-tuning. | Moderate |
| Kresevic et al., 2024 | Robust proof-of-concept with ablation design across 5 experimental setups; accuracy assessed by two blinded hepatologists with consensus; limitations include simulated environment (not clinical practice) and single model tested (GPT-4 Turbo). | Results clearly presented with accuracy gains (43% → 99%, p<0.001), breakdown by text/table/clinical scenario questions, and similarity metrics (BLEU, ROUGE, METEOR, custom score). Precision supported by repeated queries and expert adjudication. | Findings highly relevant to improving guideline interpretation in hepatology; generalizability limited to HCV guidelines, single LLM, and technical setup requiring reformatting of guideline text. | High |
| Li, 2025 | Comparative evaluation of ChatGPT-3.5, ChatGPT-4.0, and Gemini using 64 HBV-related questions. Independent grading by two physicians with a third adjudicator ensured reliability. Limitations include single-country setting and absence of patient raters. | Results clearly presented with accuracy rates by model and question type, as well as readability scores. Appropriate statistical comparisons used, though no confidence intervals reported. | Findings relevant for clinician and patient information needs in HBV, but limited by Chinese-only context, restricted rater pool, and high reading levels reducing patient applicability. | Moderate |
| Niriella et al., 2025 | Cross-sectional comparison of 20 FAQs answered by ChatGPT-3.5, Gemini, and human experts. Three blinded gastroenterologists independently rated responses. Strengths: blinded expert raters, use of statistical tests (Kruskal–Wallis). Limitations: small FAQ sample, baseline versions of LLMs only, possible bias in FAQ selection. | Results reported with domain scores (accuracy, completeness, quality) and p-values. No statistical difference between AI and experts. No confidence intervals reported, modest number of raters. | Relevant for patient education and public health communication; limited by Sri Lankan setting, English-only FAQs, baseline LLM versions, and lack of diverse patient perspectives. | Moderate |
| Pugliese et al., 2024 | Strong design with blinded expert ratings across multiple Italian centers; inclusion of patient advocate adds perspective. Limitations: subjective Likert scoring, poor inter-rater agreement (Fleiss’s kappa ~0.0), reliance on ChatGPT-3.5 only. | Results reported clearly with mean Likert scores for accuracy (4.57/6), completeness (2.14/3), and comprehensibility (2.91/3); variability across domains noted. Lack of CIs reduces precision. | Findings relevant to MASLD patient counselling and multilingual contexts, but limited by Italian-only setting, single chatbot version, and modest sample of experts. | Moderate |
| Wu et al., 2025 | Cross-sectional analysis of NHANES 2017–2018 with GPT-3.5, GPT-4, GPT-4V. Strengths: large representative dataset, comparison against validated indices, AUROC with CIs reported. Limitations: retrospective design, no biopsy/FibroScan gold standard, GPT-4V exploratory with limited accuracy, no longitudinal outcomes. | Results clearly reported with AUROC for GPT-4 (.831, 95% CI .796–.867), comparable to FLI (.817) and USFLI (.827). Correlation coefficients provided; GPT-4 outperformed GPT-3.5. GPT-4V potential noted but weaker than radiologists. | Relevant for MASLD risk stratification in population studies; generalizability supported by use of NHANES, though limited by lack of histology/imaging validation and reliance on U.S. cohort only. | Moderate |
| Yeo et al., 2023 | Robust design with independent grading by two hepatologists and resolution by a third reviewer; reproducibility tested; limitations include reliance on questions curated from social media and society FAQs, and reviewers were not blinded to ChatGPT origin | Results reported with percentages across domains; clear breakdown by category (basic knowledge, treatment, diagnosis, preventive medicine); some uncertainty due to inter-reviewer variability (20–25%) | Study is relevant to patient education and clinician support, but findings limited by single ChatGPT version (Dec 2022), U.S./U.K. context, and lack of generalizability across guideline regions | Moderate |

**Table S5**. Narrative Appraisal of Conceptual and Editorial Studies

| Study | Methodological Rigor | Clarity and Transparency | Relevance and Contribution |
| --- | --- | --- | --- |
| Berry, 2025 | Provides a structured conceptual framework for integrating LLMs into gastroenterology research, with attention to ethical use, transparency, and interdisciplinary collaboration. No empirical data, so rigor is theoretical. | Clearly organized, outlines components of the framework, but lacks methodological validation. | Highly relevant for shaping future gastroenterology research using LLMs; contribution lies in conceptual guidance rather than empirical findings. |
| Bhala, 2024 | \| Narrative expert review without systematic methodology; selective referencing but provides structured discussion of AI/LLMs in ALD. \| \| --- \| | \| Clear “Key Points” and background sections; arguments transparent and logically developed. \| \| --- \| | \| Highly relevant to alcohol-associated liver disease and the integration of AI/LLMs in clinical hepatology; emphasizes both opportunities and ethical challenges. \| \| --- \| |
| Giuffrè, 2025 (editorial) | Provides critical appraisal of a prior study on ChatGPT in GI diseases; highlights limitations in defining “accuracy” and stresses the need to use domain-knowledge (guidelines, experts) as gold standard; not empirical, but reasoning is rigorous. | Clearly written, transparent about rationale and examples (eg, Rome IV IBS criteria, privacy risks); structured argument but limited by format of a short letter. | Highly relevant to methodological standards for evaluating LLMs in gastroenterology; contributes to ethical and safety discussions, especially around accuracy definition and patient privacy. |
| Pugliese, 2025 (editorial) | Based on expert opinion and synthesis of recent studies; selective citation without systematic approach; appropriate for editorial standards but not empirical. | Well-written with structured arguments highlighting opportunities and risks; clear presentation but lacking methodological detail. | Highly relevant to MASLD clinical practice and the evolving role of LLMs; frames future research directions including integration with RAG and ethical safeguards. |

**Supplementary Table S6.** Large language models evaluated in empirical studies included in this systematic review

| Study | LLM(s) evaluated | Model configuration/family | Chronic liver disease application | Reported task-specific advantages | Key cautions for interpretation |
| --- | --- | --- | --- | --- | --- |
| Bhala, 2024 | GPT-4o with retrieval-augmented generation; custom GPT | GPT-4o; retrieval-augmented system; customized GPT | Alcohol-associated liver disease clinical reasoning | GPT-4o with retrieval augmentation showed the strongest performance in the study, approaching expert-level responses and exceeding the customized GPT model on the evaluated question set | Pilot comparison with a very small dataset; performance varied by language; findings should not be generalized beyond the specific tasks assessed |
| Colapietro, 2025 | GPT-4 | GPT-4 | Autoimmune hepatitis question answering | Responses were rated favorably for correctness, completeness, clarity, and guideline consistency in answering autoimmune hepatitis questions | Very small sample; subjective scoring; English-only evaluation; focused on a narrow disease context |
| Far, 2025 | GPT-4 | GPT-4 | Identification of cirrhosis and cirrhosis-related complications from discharge summaries | Demonstrated high accuracy for identifying cirrhosis from free-text clinical documentation and showed potential for text-based case classification in chronic liver disease research | Retrospective study; performance for complications was less robust than for cirrhosis itself; no prospective clinical workflow validation |
| Giuffre, 2025 | GPT-4 | GPT-4 within an agent-based framework | Automated treatment recommendation for chronic hepatitis C | Produced guideline-consistent treatment recommendations and supported multistep clinical decision support | Framework-based evaluation with limited real-world validation; mostly simulated or retrospective context; applicability to broader hepatology workflows remains uncertain |
| Huang, 2024 | GPT-4 | GPT-4 | Diagnosis of small hepatocellular carcinoma using CEUS LI-RADS-based imaging interpretation | Showed high concordance with radiologists in categorizing small nodules and suggested potential utility in surveillance-related interpretation | Retrospective design; dependent on report quality and lesion characteristics; requires external and prospective validation |
| Kresevic, 2024 | GPT-4 | GPT-4 with retrieval augmentation versus zero-shot prompting | Guideline-based clinical decision support for chronic hepatitis C | Retrieval augmentation improved the accuracy and relevance of guideline interpretation compared with zero-shot prompting, suggesting value for knowledge-grounded hepatology decision support | No patient-level clinical validation; findings depend on the underlying guideline database and retrieval pipeline |
| Laohawetwanit, 2025 | GPT-4 / GPT-4 Vision* | GPT-family model; vision-enabled GPT model* | Fibrosis staging in metabolic dysfunction-associated steatohepatitis | Reported strong agreement with pathologists, particularly for advanced fibrosis staging, supporting the promise of multimodal large language model use in pathology-based assessment | Retrospective study; results may be influenced by image quality and workflow variability; *model naming should be standardized against the source study before submission* |
| Li, 2025 | ChatGPT-3.5, GPT-4, Google Gemini | GPT-family and Gemini-family models | Chronic hepatitis B question answering and patient information | GPT-4 achieved the highest objective and subjective performance among the compared models and appeared particularly strong on diagnosis-related questions | No real patient interaction; readability remained above recommended patient level; comparison limited to a specific hepatitis B question set |
| Niriella, 2025 | Early ChatGPT; Google Bard | Early GPT-family model; Bard-family model | General liver disease frequently asked questions | Freely accessible general-purpose models provided a substantial proportion of correct responses and appeared more useful for general than technical questions | Accuracy declined for more complex content; subjective scoring; baseline public models only; no patient testing |
| Panzeri, 2025 | GPT-4 Vision | Vision-enabled GPT model | Fibrosis staging in metabolic dysfunction-associated steatohepatitis from biopsy images | Achieved accuracy comparable to expert pathologists when selected images were used, and performance improved further with in-context learning | Small sample; performance depended heavily on image selection and contextual prompting; weaker performance in some advanced-stage assessments |
| Sheng, 2025 | GPT-4o; Google Gemini* | GPT-4o and Gemini-family models* | Focal liver lesion diagnosis from CT/MRI report interpretation | The evaluated models showed diagnostic performance approaching that of radiologists, supporting possible use in structured imaging-report interpretation | Retrospective text-based evaluation; tertiary-center bias; *model names and findings should be cross-checked for consistency before submission* |
| Wu, 2025 | ChatGPT-3.5, GPT-4, GPT-4 Vision | GPT-family text and vision-enabled models | Diagnosis of metabolic dysfunction-associated steatotic liver disease using textual and ultrasound-image inputs | Supported integration of clinical and laboratory data for metabolic dysfunction-associated steatotic liver disease assessment, with newer GPT-4-based models appearing to perform more strongly than earlier variants within the study | Retrospective or cross-sectional evaluation; input-dependent performance; moderate sample size; no longitudinal validation |
| Yeo, 2023 | ChatGPT-3.5 | ChatGPT-3.5 | Cirrhosis and hepatocellular carcinoma question answering | Performed reasonably well for general patient-facing questions and showed utility for broad informational support | Lower performance on complex clinical questions; risk of outdated information; no real-time or clinical workflow testing |
| Zhang, 2025 | GPT-4; Google Bard | GPT-family and Bard-family models | Fatty liver disease severity grading | GPT-4 appeared to perform somewhat better than Google Bard on nuanced reasoning tasks in the evaluated vignette-based setting | Small vignette-based comparison; limited quantitative reporting; findings should be interpreted cautiously |

**Abbreviations:** CEUS LI-RADS, contrast-enhanced ultrasound Liver Imaging Reporting and Data System; GPT, generative pretrained transformer.

**Table note:** Only empirical studies were included in this summary table. Reported advantages reflect findings within individual studies and should not be interpreted as standardized head-to-head comparisons across the literature. Because included studies differed substantially in task design, datasets, languages, prompts, and outcome measures, broad claims regarding overall model superiority should be avoided.
